# Accuracy of physical examination for chronic lumbar radiculopathy

**DOI:** 10.1186/1471-2474-14-206

**Published:** 2013-07-09

**Authors:** Trond Iversen, Tore K Solberg, Bertil Romner, Tom Wilsgaard, Øystein Nygaard, Knut Waterloo, Jens Ivar Brox, Tor Ingebrigtsen

**Affiliations:** 1 Bindal Legekontor, Terråk, Norway; 2 Department of Physical Medicine and Rehabilitation, University Hospital of North Norway, Tromsø, Norway; 3 Department of Ophthalmology and Neurosurgery, University Hospital of North Norway, Tromsø, Norway; 4 The Norwegian Registry for Spine Surgery (NORspine), North Norway Regional Health Authority, Tromsø, Norway; 5 Department of Clinical Medicine, Faculty of Health Sciences, University of Tromsø, Tromsø, Norway; 6 Department of Neurosurgery, The Neuroscience Centre, Rigshospitalet, Copenhagen, Denmark; 7 Department of Community Medicine, Faculty of Health Sciences, University of Tromsø, Tromsø, Norway; 8 Department of Neuroscience, Norwegian University of Science and Technology, Trondheim, Norway; 9 Department of Neurosurgery, St Olavs University Hospital, Trondheim, Norway; 10 Department of Neurology, University Hospital of North Norway, Tromsø, Norway; 11 Department of Psychology, Faculty of Health Sciences, University of Tromsø, Tromsø, Norway; 12 Section for Back Surgery, Orthopaedic Department, Oslo University Hospital, Oslo, Norway; 13 Centre for Clinical Governance Research, Australian Institute of Health Innovation, University of New South Wales, Sydney, NSW, Australia

**Keywords:** Sensitivity, Accuracy, Likelihood ratio, Lumbar radiculopathy, Physical examination

## Abstract

**Background:**

Clinical examination of patients with chronic lumbar radiculopathy aims to clarify whether there is nerve root impingement. The aims of this study were to investigate the association between findings at clinical examination and nerve root impingement, to evaluate the accuracy of clinical index tests in a specialised care setting, and to see whether imaging clarifies the cause of chronic radicular pain.

**Methods:**

A total of 116 patients referred with symptoms of lumbar radiculopathy lasting more than 12 weeks and at least one positive index test were included. The tests were the straight leg raising test, and tests for motor muscle strength, dermatome sensory loss, and reflex impairment. Magnetic resonance imaging (n = 109) or computer tomography (n = 7) were imaging reference standards. Images were analysed at the level of single nerve root(s), and nerve root impingement was classified as present or absent. Sensitivities, specificities, and positive and negative likelihood ratios (LR) for detection of nerve root impingement were calculated for each individual index test. An overall clinical evaluation, concluding on the level and side of the radiculopathy, was performed.

**Results:**

The prevalence of disc herniation was 77.8%. The diagnostic accuracy of individual index tests was low with no tests reaching positive LR >4.0 or negative LR <0.4. The overall clinical evaluation was slightly more accurate, with a positive LR of 6.28 (95% CI 1.06–37.21) for L4, 1.74 (95% CI 1.04–2.93) for L5, and 1.29 (95% CI 0.97–1.72) for S1 nerve root impingement. An overall clinical evaluation, concluding on the level and side of the radiculopathy was also performed, and receiver operating characteristic (ROC) analysis with area under the curve (AUC) calculation for diagnostic accuracy of this evaluation was performed.

**Conclusions:**

The accuracy of individual clinical index tests used to predict imaging findings of nerve root impingement in patients with chronic lumbar radiculopathy is low when applied in specialised care, but clinicians’ overall evaluation improves diagnostic accuracy slightly. The tests are not very helpful in clarifying the cause of radicular pain, and are therefore inaccurate for guidance in the diagnostic workup of the patients. The study population was highly selected and therefore the results from this study should not be generalised to unselected patient populations in primary care nor to even more selected surgical populations.

## Background

Lumbar radiculopathy is a common reason for physician consultations and imaging referrals [1-3]. Typical symptoms are radiating pain, often with numbness, paraesthesia, and/or muscle weakness [1,4]. Clinical examination aims to clarify whether there is mechanical impingement of a nerve root [5]. The most common clinical diagnostic tests are the straight leg raising test, and tests for tendon reflexes, motor weakness, and sensory deficits [6]. An inaccurate clinical diagnosis may lead to unnecessary imaging and healthcare expenditure, and additional concerns for patients [7-12].

The aim with imaging is to confirm or disprove a clinical suspicion, and to provide a roadmap for planning of surgical or other intervention procedures, if indicated. Mechanical nerve root impingements demonstrated with magnetic resonance imaging (MRI) or computer tomography (CT) is an accepted reference standard [13].

Systematic reviews on the diagnostic properties of clinical diagnostic tests for lumbar radiculopathy report variable accuracy, with sensitivities ranging from 0.14 to 0.61 for sensory deficits and impaired tendon reflexes [14,15], 0.27 to 0.62 for motor weakness [14,16], and 0.35 to 0.81 for the straight leg raising test [17]. Most studies report likelihood ratios (LRs) suggesting negligible differences between pre- and post-test probabilities for presence of nerve root impingement as the target condition, indicating limited value of the tests in clinical decision-making. A recent Cochrane review confirmed poor diagnostic performance of diagnostic tests in 18 studies from specialised care [13].

This review raised concern that none of the reported studies specifically discriminated between nerve root impingement and just the presence of a disc herniation when using imaging as a reference standard. This could be a major bias, since the prevalence of disc bulging or herniation in unselected populations without radiculopathy symptoms is high [18].

The aims of this study are to investigate the association between findings at clinical examination and nerve root impingement, to evaluate the accuracy of clinical index tests in a specialised care setting, and to see whether imaging clarifies the cause of chronic radicular pain.

## Methods

### Study participants

The study was performed as part of a multicentre randomised controlled trial on the treatment effect of caudal epidural injections [19]. Eligible patients with suspected chronic lumbar radiculopathy, aged between 20 and 60 years, referred to outpatient multidisciplinary back clinics of five Norwegian hospitals, were consecutively assessed for inclusion. All patients were referred with a history suggesting chronic lumbar radiculopathy, and the clinical diagnosis was verified with at least one corresponding positive clinical test (index test) consistent with affection of a specific lumbar nerve root. These inclusion criteria ensured a homogenous patient population with clinically verified lumbar radiculopathy and a high pre-test probability of nerve root impingement. MRI or CT was used to specifically clarify whether the nerve root in question was impinged or not. The reference standard was set to be disc herniation causing impingement (compression and/or dislocation) of a spinal nerve root. Written informed consent was obtained, and the Regional Committee for Medical and Health Research Ethics in North Norway approved the study.

**Figure 1:**
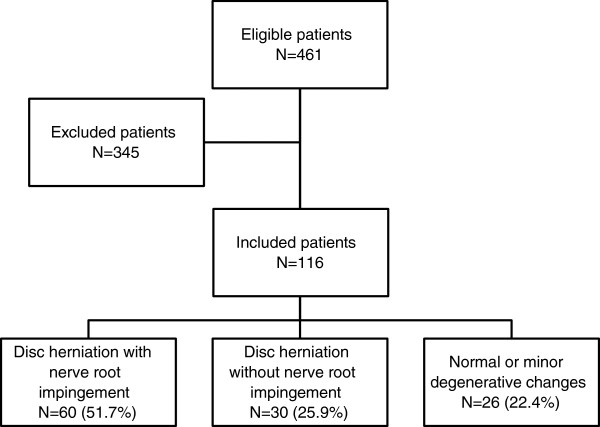
Flowchart showing number of eligible and excluded patients, and results from MRI or CT in the 116 included patients.

We assessed 461 patients with suspected lumbar radiculopathy for inclusion (Figure 1). 376 (81.6%) were referred from general practitioners, and 85 (18.4%) were internally referred in the participating hospitals. The inclusion criteria were unilateral lumbar radiculopathy lasting for more than 12 weeks and one or more positive index tests consistent with nerve root affection. The intensity of the leg pain, radiating from the back to below the knee, had to be comparable to or worse than the back pain. Whilst obtaining the patient’s history, enquiries were made about the intensity of leg and low back pain on a visual analogue scale, the possible dermatome distribution of the pain, the presence of paraesthesia in the leg, whether the pain was aggravated by forward flexion or sitting, and whether there was any muscle weakness in the lower extremity.

We excluded 345 (74.8%) patients fulfilling predefined exclusion criteria according to the original randomised control trial [19]: 146 (42.3%) due to unspecific low back pain with referred leg pain, 105 (30.4%) due to radiculopathy improving during the last two weeks, 24 (7.0%) due to radiculopathy requiring referral to surgery, 16 (4.6%) because of earlier back surgery, 37 (10.7%) due to different medical conditions (pregnancy, breast feeding, use of anticlotting medication), and 17 (4.9%) because they declined to participate.

### Physical examination

The physical examination was performed according to the recommendations given by the American Spinal Injury Association [20-22]. It consisted of the following index tests: the straight leg raising test, the femoral nerve stretch test, testing of muscle power in seven muscle groups on a five-point scale, dermatome sensory loss using light touch and pin prick classified on a three-point scale, and reflex impairment testing on a four-point scale. Each index test was dichotomised as being normal or abnormal according to the standard neurological classification. The straight leg raising test was considered abnormal when pain occurred before 60 degrees passive elevation from horizontal, and the femoral nerve stretch test was considered positive when the patient experienced radiating pain [23].

Specialists in neurology or physical medicine and rehabilitation did the examination in cooperation with a physiotherapist. Prior to the study, they were trained to perform the tests in a standardised way.

Based on an overall evaluation of the patient history and results of all the index tests, a clinical decision was reached for each patient concerning the suspected level and side of nerve root affection [24-27]. The clinical decision for a nerve root involvement required a history of radicular pain accompanied by one or more corresponding positive index tests. The clinicians were blinded to the results of the imaging until this decision had been reached. To diagnose an L4 radiculopathy the clinician placed emphasis on the femoral nerve stretch test, the straight leg raise test, the knee reflex, sensory loss in the L4 dermatome and the muscle power for the ankle dorsiflexion. To diagnose an L5 radiculopathy the clinician focused on the straight leg raise test, sensory loss in the L5 dermatome, and the muscle power for the hip abduction, ankle dorsiflexion, ankle eversion, and the big toe extension. For an S1 radiculopathy the clinician emphasized the straight leg raise test, the ankle reflex, sensory loss in the S1 dermatome, and the muscle power for hip extension, knee flexion, ankle plantarflexion, and ankle eversion.

### Imaging reference standard

MRI in 109 (94.0%) patients or CT in 7 (6.0%) patients was performed. Experienced radiologists evaluated the images, and a written report from the radiologists was available for the clinicians to be able to exclude patients with severe intra-spinal pathology obviously demanding surgery [19,28].

All the MRI and CT scans were re-evaluated by two independent neuroradiologists using the Nordic Modic Classification [29]. They were blinded regarding patient history and clinical findings. The locations of the disc herniation were identified in the axial plane, and were categorised as being localised centrally or to the left or right in the spinal canal [30]. In cases of disagreement, a consensus was reached emphasising the most experienced.

### Statistical analysis

We calculated means and standard deviations (SD) for continuous variables, and frequencies and proportions for categorical variables. The prevalence of nerve root impingement based on the reference standard and the post-test probabilities for a positive and negative test were calculated. Diagnostic accuracy was quantified by calculating sensitivities, specificities, and positive and negative likelihood ratios (LR), including 95% confidence intervals (CI), for each clinical test. In a multivariable logistic regression model we included all index tests as independent variables. The estimated model was used to predict the probability of a positive MRI/CT for each patient. These probabilities were used to produce a receiver operating characteristic (ROC) curve and an estimate for the area under the curve (AUC). All analyses were performed using the Statistical Package for the Social Sciences software (SPSS), version 19 (IBM Software, NY, USA).

## Results

In total, 116 patients with unilateral chronic lumbar radiculopathy were included. Their clinical and demographic characteristics are summarised in Table 1. Mean age was 42.0 (SD 10.3) years, 68 (58.6%) were males, and the mean duration of symptoms on inclusion was 42.0 (SD 99.0) weeks. Figure 1 shows the results of MRI or CT for the included patients. The overall prevalence of disc herniation at any of the studied lumbar levels (L2 to S1) was 77.8%.

**Table 1:** Clinical and demographic characteristics of 116 patients with chronic lumbar radiculopathy

**Characteristics**	
Smoker	49 (42.2)
Body mass index (kg/m^2^) Mean (SD)	26.3 (3.8)
Physically demanding work	58 (50.0)
**Educational level**	
Secondary school	94 (81.0)
College/University	24 (19.0)
Receiving sickness benefit	53 (45.7)
VAS Low back pain (0–100) Mean (SD)	47.6 (24.3)
VAS Leg pain (0–100) Mean (SD)	50.6 (24.7)
Time from referral to inclusion (weeks) Mean (SD)	6.4 (6.8)

Data are number (%) unless stated otherwise.

*SD* Standard Deviation.

*VAS* Visual Analogue Scale.

Table 2 shows the frequencies of positive index tests, the overall clinical evaluation, and the imaging findings. Table 3 shows the diagnostic accuracies for the different index tests for detection of the level and side of the nerve root impingement. None of the individual tests were highly accurate, as both sensitivities and specificities were low with wide CIs. All positive LRs were ≤4.0, and all negative LRs ≥0.4.

**Table 2:** Incidence of positive index and reference tests in painful leg*

**Index test or reference test**	**Positive**	**Percent**
**Nerve stretch tests**		
Femoral nerve stretch test	6	6.0
Straight leg raise test	62	53.4
**Reflex tests**		
Knee reflex	21	18.1
Ankle reflex	47	40.5
**Sensory loss testing**		
L3	4	3.4
L4	14	12.1
L5	31	26.7
S1	52	44.8
**Motor strength/weakness**		
Hip flexion (Iliopsoas L1,L2,L3)	13	11.2
Hip extension (Gluteus maximus L5,S1,S2)	14	12.1
Hip abduction (Gluteus medius L4,L5,S1)	9	7.7
Knee flexion (Hamstrings L5,S1,S2)	64	55.2
Knee extension (Quadriceps femoris L2,L3,L4)	1	0.9
Ankle dorsiflexion (Tibialis anterior L4,L5)	37	31.9
Ankle plantarflexion (Gastro-cnemius and Soleus S1,S2)	45	3.9
Ankle eversion (Peronei L5,S1)	80	6.9
Big toe extension (Extensor hallucis longus L5,S1)	25	21.5
**Clinician suspected spinal nerve root impingement**		
L3	1	0.9
L4	7	6.0
L5	37	31.9
S1	71	61.2
**MRI or CT proven disc herniation with spinal nerve root impingement**		
L3	0	0
L4	3	2.6
L5	30	25.9
S1	27	23.3
**MRI or CT proven disc herniation without spinal nerve root impingement**		
L3	0	0
L4	1	0.9
L5	12	10.3
S1	17	14.6
**MRI or CT normal or with minor degenerative changes without spinal nerve root impingement**		
All lumbar spinal levels	26	22.4

*Number of patients 116.

**Table 3:** Diagnostic accuracy of individual neurological tests

**Predictor**	**L4 nerve root impingement**				**L5 nerve root impingement**				**S1 nerve root impingement**			
	**Sens**	**Spec**	**+LR**	**−LR**	**Sens**	**Spec**	**+LR**	**−LR**	**Sens**	**Spec**	**+LR**	**−LR**
**Femoral nerve stretch test**	*	*	*	*	0.17 (0.07–0.33)	0.99 (0.94–1.00)	14.33 (1.74–117.80)	0.84 (0.72–0.99)	*	*	*	*
**Straight leg raise test**	*	*	*	*	0.53 (0.36–0.70)	0.47 (0.36–0.57)	1.00 (0.68–1.47)	1.00 (0.64–1.57)	0.63 (0.44–0.78)	0.49 (0.39–0.60)	1.24 (0.87–1.78)	0.75 (0.44–1.28)
**Knee reflex**	0.67 (0.21–0.94)	0.83 (0.75–0.89)	3.96 (1.61–9.74)	0.40 (0.08–1.99)	0.18 (0.08–0.37)	0.75 (0.63–0.84)	0.73 (0.30–1.79)	1.09 (0.87–1.37)	0.11 (0.04–0.28)	0.80 (0.70–0.87)	0.55 (0.18–1.72)	1.11 (0.94–1.32)
**Ankle reflex**	0.67 (0.21–0.94)	0.60 (0.51–0.69)	1.67 (0.73–3.84)	0.55 (0.11–2.76)	0.27 (0.14–0.44)	0.55 (0.44–0.65)	0.59 (0.31–1.11)	1.34 (1.00–1.79)	0.44 (0.27–0.639	0.61 (0.50–0.70)	1.13 (0.69–1.85)	0.92 (0.63–1.33)
**Sensory loss L4**	0.33 (0.06–0.79)	0.88 (0.81–0.93)	2.90 (0.54–15.55)	0.75 (0.34–1.68)	0.20 (0.10–0.37)	0.91 (0.83–0.95)	2.15 (0.81–5.70)	0.88 (0.73–1.07)	0.11 (0.04–0.28)	0.88 (0.79–0.93)	0.90 (0.27–2.99)	1.01 (0.87–1.18)
**Sensory loss L5**	0.33 (0.06–0.79)	0.73 (0.65–0.81)	1.26 (0.25–6.40)	0.91 (0.40–2.03)	0.43 (0.27–0.61)	0.79 (0.69–0.86)	2.07 (1.16–3.70)	0.72 (0.51–1.00)	0.18 (0.08–0.37)	0.71 (0.61–0.79)	0.63 (0.27–1.49)	1.15 (0.92–1.44)
**Sensory loss S1**	*	*	*	*	0.33 (0.19–0.51)	0.51 (0.41–0.61)	0.68 (0.39–1.18)	1.30 (0.94–1.81)	0.44 (0.27–0.63)	0.55 (0.45–0.65)	0.99 (0.61–1.60)	1.01 (0.69–1.48)
**Hip flexion**	*	*	*	*	0.23 (0.12–0.41)	0.93 (0.86–0.97)	3.34 (1.22–9.16)	0.82 (0.67–1.01)	*	*	*	*
**Hip extension**	0.33 (0.06–0.79)	0.88 (0.81–0.93)	2.90 (0.54–15.55)	0.75 (0.34–1.68)	0.03 (0.01–0.17)	0.85 (0.76–0.91)	0.22 (0.03–1.61)	1.14 (1.02–1.27)	0.18 (0.08–0.37)	0.90 (0.82–0.94)	1.83 (0.67–5.00)	0.91 (0.75–1.10)
**Hip abduction**	*	*	*	*	0.07 (0.02–0.21)	0.92 (0.84–0.96)	0.82 (0.18–3.73)	1.01 (0.91–1.13)	0.04 (0.01–0.18)	0.91 (0.83–0.95)	0.41 (0.05–3.15)	1.06 (0.96–1.17)
**Knee flexion**	0.67 (0.21–0.94)	0.45 (0.36–0.54)	1.22 (0.54–2.75)	0.74 (0.15–3.71)	0.50 (0.33–0.67)	0.43 (0.33–0.53)	0.88 (0.59–1.31)	1.16 (0.75–1.79)	0.70 (0.51–0.84)	0.49 (0.39–0.60)	1.39 (1.01–1.92)	0.60 (0.32–1.11)
**Knee extension**	*	*	*	*	*	*	*	*	*	*	*	*
**Ankle dorsiflexion**	0.33 (0.06–0.79)	0.68 (0.59–0.76)	1.05 (0.20–5.30)	0.98 (0.44–2.20)	0.40 (0.25–0.58)	0.71 (0.61–0.79)	1.38 (0.80–2.38)	0.85 (0.61–1.17)	0.26 (0.13–0.45)	0.66 (0.56–0.75)	0.77 (0.38–1.55)	1.12 (0.85–1.46)
**Ankle plantarflexion**	0.67 (0.21–0.94)	0.62 (0.53–0.70)	1.75 (0.76–4.03)	0.54 (0.11–2.68)	0.27 (0.14–0.44)	0.57 (0.46–0.67)	0.62 (0.33–1.18)	1.29 (0.97–1.71)	0.44 (0.27–0.63)	0.63 (0.52–0.72)	1.20 (0.73–1.98)	0.88 (0.61–1.28)
**Ankle eversion**	0.67 (0.21–0.94)	0.31 (0.23–0.40)	0.97 (0.43–2.17)	1.08 (0.21–5.46)	0.45 (0.27–0.65)	0.28 (0.19–0.38)	0.63 (0.39–1.01)	1.96 (1.17–3.26)	0.70 (0.51–0.84)	0.31 (0.23–0.42)	1.03 (0.77–1.36)	0.94 (0.49–1.82)
**Big toe extension**	*	*	*	*	0.33 (0.19–0.51)	0.83 (0.73–0.89)	1.91 (0.97–3.79)	0.81 (0.62–1.06)	0.15 (0.06–0.32)	0.76 (0.67–0.84)	0.63 (0.24–1.67)	1.11 (0.49–1.82)

Values in each cell are estimates and 95% confidence intervals.

Sens indicates sensitivity (TP/TP+FN).

Spec indicates specificity (TN/TN+FP).

+LR indicates positive likelihood ratio (Sens/1-Spec).

−LR indicates negative likelihood ratio (1-Sens/Spec).

*No TP (True Positive).

Table 4 shows that the clinicians’ overall evaluations using information from all relevant index tests to predict nerve root impingement were slightly more accurate than each of the individual index tests. ROC analysis of the diagnostic properties of the overall clinical evaluations showed AUCs of 0.95 (95% CI 0.90–1.00) for L4, 0.67 (95% CI 0.56–0.77) for L5, and 0.66 (95% CI 0.54–0.77) for S1 nerve root impingement.

**Table 4:** Diagnostic accuracy of clinician examination conclusion

**Predictor**	**L4 nerve root impingement**				**L5 nerve root impingement**				**S1 nerve root impingement**			
	**Sens**	**Spec**	**+LR**	**−LR**	**Sens**	**Spec**	**+LR**	**−LR**	**Sens**	**Spec**	**+LR**	**−LR**
**Clinician concluded L4 nerve root impingement**	0.33 (0.06–0.79)	0.95 (0.89–0.97)	6.28 (1.06–37.21)	0.70 (0.32–1.57)	0.10 (0.03–0.26)	0.95 (0.89–0.98)	2.15 (0.51–9.06)	0.94 (0.83–1.07)	*	*	*	*
**Clinician concluded L5 nerve root impingement**	0.33 (0.06–0.79)	0.68 (0.59–0.76)	1.05 (0.21–5.30)	0.98 (0.43–2.20)	0.47 (0.30–0.64)	0.73 (0.63–0.81)	1.74 (1.04–2.93)	0.73 (0.51–1.04)	0.26 (0.13–0.45)	0.66 (0.56–0.75)	0.77 (0.38–1.55)	1.12 (0.85–1.46)
**Clinician concluded S1 nerve root impingement**	0.33 (0.06–0.79)	0.38 (0.30–0.47)	0.54 (0.11–2.68)	1.75 (0.76–4.03)	0.43 (0.27–0.61)	0.32 (0.23–0.43)	0.64 (0.42–0.99)	1.74 (1.12–2.69)	0.74 (0.55–0.87)	0.43 (0.33–0.53)	1.29 (0.97–1.72)	0.61 (0.31–1.20)

Values in each cell are an estimate and 95% confidence intervals.

Sens indicates sensitivity (TP/TP+FN).

Spec indicates specificity (TN/TN+FP).

+LR indicates positive likelihood ratio (Sens/1-Spec).

−LR indicates negative likelihood ratio (1-Sens/Spec).

*No TP (True Positive).

## Discussion

This study included patients with symptoms suggesting lumbar radiculopathy. Patients were recruited by screening and referral from general practitioners, and those with large disc herniation obviously requiring surgery were excluded. The sample emerging from these criteria is typical for the chronic radiculopathy population seen in specialised care. Results from the study are relevant for our understanding of diagnostic accuracy in the common clinical setting where specialists have access to imaging findings prior to the clinical examination, and often are challenged by having to evaluate which of numerous positive imaging findings are to be considered clinically relevant.

The main finding is that individual clinical index tests lack diagnostic accuracy for predicting whether a lumbar nerve root is impinged or not at a specific level in patients with chronic lumbar radiculopathy in specialised care. The overall clinical evaluation, consisting of the specialists’ combined interpretation of the patients’ history and all index tests, was somewhat more accurate. For L5 and S1 nerve root impingement, however, LRs did not reach the levels usually considered necessary to influence post-test probability and thereby clinical decision-making (positive LR >5.0 and negative LR <0.2) [31]. Accuracy was better (positive LR 6.28, negative LR 0.70) for L4 nerve root impingement. This was probably because L4 nerve root involvement occurred only in 3 (2.6%) cases, and was suspected after the overall clinical evaluation only in 7 (6.0%) cases. This resulted in a high number of true negatives, and thereby high specificity. Clinically, the low pre-test probability for L4 nerve root involvement is well known [32], and these test properties are therefore not very useful. Accordingly, clinical examination is inaccurate both for predicting the presence or absence of nerve root impingement, and for clarifying the relevant level and side in patients with multiple positive imaging findings.

Our findings are mainly in accordance with other studies of selected populations from specialised care [13]. Most previous studies have, however, aimed for a generalised understanding of test properties from such selected materials [13]. This approach is confusing, as the pre-test probability always must be taken into consideration. Recently, a study aimed to specifically investigate the accuracy of clinical index tests from the neurological examination for identification of the level of disc herniation in patients with the target condition already confirmed by MRI [33]. Unfortunately the study did not find evidence to support this. The results were disappointing, with no single test reaching an AUC >0.75, and only slightly better results (AUC = 0.80) for the neurologists’ overall evaluation.

It has been a weakness of most previous studies that interpretation of the imaging findings has been limited to categorising the target condition (usually a disc herniation) as present or not, without considering whether a nerve root actually was impinged at the relevant spinal level and side [34]. We therefore improved the study design by specifically addressing findings relevant for clinical decision-making: correspondence between index tests and impingement of specific nerve roots as revealed by MRI [32]. Disappointingly, this did not improve diagnostic accuracy, neither for individual tests nor for the clinicians’ overall evaluation. AUCs for L5 and S1 nerve root impingement did not reach levels above 0.66, which are even lower than those observed by Hancock et al. in an almost similar specialised care setting [33]. This could be because we used one or more positive index tests as an inclusion criterion, which probably increased both the proportion of false positives and false negatives. The false negatives increased because the index tests are not independent of each other, implying that inclusion based on one or more positive tests entails an increased proportion of false negatives, since many tests are performed in each patient. We do not consider the selection of patients in our study a methodological weakness, but rather an expression of clinical reality in specialised care. There should, however, be concern about both the definition of the target condition and the reference standard being subjects to bias. First, neuroanatomical overlap between spinal segments influences accuracy when the analysis is done on the level of each single nerve root [35-37]. Patients may have radiculopathy from causes other than ongoing nerve root impingement, and even when an impingement is present, this is not necessarily the cause of the pain. Imaging showed no sign of nerve root impingement in 56 (48.3%) of the included cases despite a clear history and clinical findings suggesting lumbar radiculopathy. This confirms that radiculopathy may have other causes, such as neuropathic and inflammatory conditions, or be mimicked by myofascial pain [6,38-40]. Moreover, disc herniation without nerve root impingement was demonstrated in 25.9% of the included patients, and in 73.8% of those excluded due to symptoms classified as unspecific low back pain with referred leg pain. This is not surprising, since the prevalence of disc herniation revealed by MRI in the general population is known to be as high as 30% [3,18,41-44].

We suggest that our findings reflect clinical reality very well: in a population selected by referral from primary care and exclusion of the most obvious surgical cases, co-morbidity bias and imaging findings not related to the symptoms are common. Diagnostic imaging combined with clinical tests is therefore inaccurate for clarifying the cause of radicular pain. This is probably one of the reasons why these patients are so difficult to treat, and the same inaccuracy may cause significant inclusion bias in clinical trials evaluating treatments for lumbar radiculopathy.

The present study has weaknesses. We did not register inter-tester variability for the clinical tests and image interpretations. However, all clinicians were trained to perform the tests in a standardised manner, and agreement should thus be superior to that achieved between clinicians in daily practice [22]. MRI was substituted with CT in 7 (6.0%) of the study subjects. A few cases of nerve root impingement may have been missed, but this is unlikely to have influenced the results significantly. Further, the duration of symptoms (average 42 weeks) was relatively long. Development of chronic centralised pain followed by regression of nerve root impingement may have occurred in some patients, and our results may not be generalisable to situations with shorter symptom duration.

Finally, it must be emphasised that the index tests work differently when applied in other settings. In unselected primary care populations, the proportion of false positives will be lower and the specificity of the tests higher. Accordingly, the tests may be useful in primary care to reduce the post-test likelihood of lumbar radiculopathy, and thereby restrict unnecessary referrals for imaging and specialised care. On the other hand, when applied in a highly selected surgical patient population with shorter duration of symptoms and a large disc herniation obviously corresponding with the symptoms, the proportion of true positives will be high and the proportion of false positives low, resulting in high sensitivity and specificity. The results from the present study should therefore not be generalised to unselected patient populations in primary care nor to even more selected surgical populations.

## Conclusions

In conclusion, the accuracy of individual clinical index tests used to predict imaging findings of nerve root impingement in patients with lumbar radiculopathy is low when applied in specialised care, and clinicians’ overall evaluation does not improve diagnostic accuracy significantly. Accordingly, the tests are not very helpful in clarifying the cause of radicular pain, and are therefore inaccurate for treatment guidance of patients who often have multiple positive imaging findings. These results suggest that previous belief in the benefit of combining different neurological tests to accurately diagnose the level of nerve root affection has been exaggerated [45,46]. Co-morbidity and imaging findings not related to the symptoms are probably the most important causes for diagnostic inaccuracy in chronic lumbar radiculopathy [3,28,39,47-49].

## Abbreviations

AUC: Area under the curve; CI: Confidence interval; CT: Computer tomography; LR: Likelihood ratio; MRI: Magnetic resonance imaging; ROC: Receiver operating characteristic; SD: Standard deviation.

## Competing interests

The authors declare that they have no competing interests.

## Authors’ contributions

TI contributed to the study design, data collection, data analysis, interpretation, and writing of the manuscript. TKS, ØN, ToI, TW, and BR contributed to the study design, data analysis, interpretation, and writing of the manuscript. JIB and KW contributed to data analysis, interpretation, and writing of the manuscript. All authors reviewed and approved the final version of the manuscript.

## Pre-publication history

The pre-publication history for this paper can be accessed here:

http://www.biomedcentral.com/1471-2474/14/206/prepub

## References

[B1] Masters S Lind R Musculoskeletal pain - presentations to general practice Aust Fam Physician 2010 39 425 428 20628683

[B2] McGuirk B Bogduk N Evidence-based care for low back pain in workers eligible for compensation Occup Med (Lond) 2007 57 36 42 17046988 10.1093/occmed/kql102

[B3] Li AL Yen D Effect of increased MRI and CT scan utilization on clinical decision-making in patients referred to a surgical clinic for back pain Can J Surg 2011 54 128 132 10.1503/cjs.001510 21443829 PMC3116695

[B4] Waddell G Main CJ Morris EW Venner RM Rae PS Sharmy SH Normality and reliability in the clinical assessment of backache Br Med J (Clin Res Ed) 1982 284 1519 1523 10.1136/bmj.284.6328.1519 PMC1498438 6211214

[B5] Janardhana AP Rajagopal Rao S Kamath A Correlation between clinical features and magnetic resonance imaging findings in lumbar disc prolapse Indian J Orthop 2010 44 263 269 10.4103/0019-5413.65148 20697478 PMC2911925

[B6] Cannon DE Dillingham TR Miao H Andary MT Pezzin LE Musculoskeletal disorders in referrals for suspected lumbosacral radiculopathy Am J Phys Med Rehabil 2007 86 957 961 10.1097/PHM.0b013e31815b614a 18090436

[B7] Atlas SJ Keller RB Wu YA Deyo RA Singer DE Long-term outcomes of surgical and nonsurgical management of sciatica secondary to a lumbar disc herniation: 10 year results from the maine lumbar spine study Spine (Phila Pa 1976) 2005 30 927 935 10.1097/01.brs.0000158954.68522.2a 15834338

[B8] Martin BI Turner JA Mirza SK Lee MJ Comstock BA Deyo RA Trends in health care expenditures, utilization, and health status among US adults with spine problems, 1997–2006 Spine (Phila Pa 1976) 2009 34 2077 2084 10.1097/BRS.0b013e3181b1fad1 19675510

[B9] Deyo RA Mirza SK Turner JA Martin BI Overtreating chronic back pain: time to back off? J Am Board Fam Med 2009 22 62 68 10.3122/jabfm.2009.01.080102 19124635 PMC2729142

[B10] Carragee EJ Clinical practice. Persistent low back pain N Engl J Med 2005 352 1891 1898 10.1056/NEJMcp042054 15872204

[B11] Daffner SD Hymanson HJ Wang JC Cost and use of conservative management of lumbar disc herniation before surgical discectomy Spine J 2010 10 463 468 10.1016/j.spinee.2010.02.005 20359960

[B12] Akobeng AK Understanding diagnostic tests 2: likelihood ratios, pre- and post-test probabilities and their use in clinical practice Acta Paediatr 2007 96 487 491 10.1111/j.1651-2227.2006.00179.x 17306009

[B13] Van Der WindtDASimonsERiphagenIIAmmendoliaCVerhagenAPLaslettMPhysical examination for lumbar radiculopathy due to disc herniation in patients with low-back painCochrane Database Syst Rev2010Issue 2, Art. No.: CD00743110.1002/14651858.CD007431.pub220166095

[B14] Vroomen PC de Krom MC Wilmink JT Kester AD Knottnerus JA Diagnostic value of history and physical examination in patients suspected of lumbosacral nerve root compression J Neurol Neurosurg Psychiatry 2002 72 630 634 10.1136/jnnp.72.5.630 11971050 PMC1737860

[B15] Albeck MJ A critical assessment of clinical diagnosis of disc herniation in patients with monoradicular sciatica Acta Neurochir (Wien) 1996 138 40 44 10.1007/BF01411722 8686523

[B16] KNUTSSON B Comparative value of electromyographic, myelographic and clinical-neurological examinations in diagnosis of lumbar root compression syndrome Acta Orthop Scand Suppl 1961 49 1 135 13757256

[B17] Scaia V Baxter D Cook C The pain provocation-based straight leg raise test for diagnosis of lumbar disc herniation, lumbar radiculopathy, and/or sciatica: a systematic review of clinical utility J Back Musculoskelet Rehabil 2012 25 215 223 23220802 10.3233/BMR-2012-0339

[B18] Borenstein DG O’Mara JW Jr Boden SD Lauerman WC Jacobson A Platenberg C The value of magnetic resonance imaging of the lumbar spine to predict low-back pain in asymptomatic subjects: a seven-year follow-up study J Bone Joint Surg Am 2001 83-A 1306 1311 11568190 10.2106/00004623-200109000-00002

[B19] Iversen T Solberg TK Romner B Wilsgaard T Twisk J Anke A Effect of caudal epidural steroid or saline injection in chronic lumbar radiculopathy: multicentre, blinded, randomised controlled trial BMJ 2011 343 d5278 10.1136/bmj.d5278 21914755 PMC3172149

[B20] Linassi G Li Pi SR Marino RJ A web-based computer program to determine the ASIA impairment classification Spinal Cord 2010 48 100 104 10.1038/sc.2009.98 19704413

[B21] Marino RJ Barros T Biering-Sorensen F Burns SP Donovan WH Graves DE International standards for neurological classification of spinal cord injury J Spinal Cord Med 2003 26 Suppl 1 S50 S56 16296564 10.1080/10790268.2003.11754575

[B22] Waring WP III Biering-Sorensen F Burns S Donovan W Graves D Jha A _ 2009 review and revisions of the international standards for the neurological classification of spinal cord injury J Spinal Cord Med 2010 33 346 352 21061894 10.1080/10790268.2010.11689712 PMC2964022

[B23] Charnley J Orthopaedic signs in the diagnosis of disc protrusion. With special reference to the straight-leg-raising test Lancet 1951 1 186 192 14795816 10.1016/s0140-6736(51)93353-3

[B24] Stanley H Orthopaedic Neurology. A Diagnostic Guide to Neurological Levels 1997 Philadelphia: Lippincott-Raven

[B25] Malanga GA Nadler SF Musculoskeletal Physical Examination. An Evidence-Based Approach 2006 Philadelphia: Mosby

[B26] Buckup K Clinical Tests for the Musculoskeletal System. Examination-Signs-Phenomenan 2008 2 Stuttgart: Thieme

[B27] Andersson GB Deyo RA History and physical examination in patients with herniated lumbar discs Spine (Phila Pa 1976) 1996 21 10S 18S 10.1097/00007632-199612151-00003 9112321

[B28] Suri P Hunter DJ Katz JN Li L Rainville J Bias in the physical examination of patients with lumbar radiculopathy BMC Musculoskelet Disord 2010 11 275 10.1186/1471-2474-11-275 21118558 PMC3009628

[B29] Jensen TS Sorensen JS Kjaer P Intra- and interobserver reproducibility of vertebral endplate signal (modic) changes in the lumbar spine: the Nordic Modic Consensus Group classification Acta Radiol 2007 48 748 754 10.1080/02841850701422112 17729006

[B30] Summers B Malhan K Cassar-Pullicino V Low back pain on passive straight leg raising: the anterior theca as a source of pain Spine (Phila Pa 1976) 2005 30 342 345 10.1097/01.brs.0000152378.93868.c8 15682017

[B31] Jaeschke R Guyatt G Sackett DL Users’ guides to the medical literature. III. How to use an article about a diagnostic test. A. Are the results of the study valid? Evidence-Based Medicine Working Group JAMA 1994 271 389 391 10.1001/jama.1994.03510290071040 8283589

[B32] Valat JP Genevay S Marty M Rozenberg S Koes B Sciatica Best Pract Res Clin Rheumatol 2010 24 241 252 10.1016/j.berh.2009.11.005 20227645

[B33] Hancock MJ Koes B Ostelo R Peul W Diagnostic accuracy of the clinical examination in identifying the level of herniation in patients with sciatica Spine (Phila Pa 1976) 2011 36 E712 E719 10.1097/BRS.0b013e3181ee7f78 21224761

[B34] Majlesi J Togay H Unalan H Toprak S The sensitivity and specificity of the Slump and the Straight Leg Raising tests in patients with lumbar disc herniation J Clin Rheumatol 2008 14 87 91 10.1097/RHU.0b013e31816b2f99 18391677

[B35] Beattie P Nelson R Clinical prediction rules: what are they and what do they tell us? Aust J Physiother 2006 52 157 163 10.1016/S0004-9514(06)70024-1 16942450

[B36] Mobbs RJ Steel TR Migration of lumbar disc herniation: an unusual case J Clin Neurosci 2007 14 581 584 10.1016/j.jocn.2006.04.002 17430782

[B37] Sucu HK Gelal F Lumbar disk herniation with contralateral symptoms Eur Spine J 2006 15 570 574 10.1007/s00586-005-0971-x 16231173 PMC3489328

[B38] Riksman JS Williamson OD Walker BF Delineating inflammatory and mechanical sub-types of low back pain: a pilot survey of fifty low back pain patients in a chiropractic setting Chiropr Man Therap 2011 19 5 10.1186/2045-709X-19-5 21299867 PMC3048575

[B39] Boden SD McCowin PR Davis DO Dina TS Mark AS Wiesel S Abnormal magnetic-resonance scans of the cervical spine in asymptomatic subjects. A prospective investigation J Bone Joint Surg Am 1990 72 1178 1184 2398088

[B40] Lauder TD Musculoskeletal disorders that frequently mimic radiculopathy Phys Med Rehabil Clin N Am 2002 13 469 485 10.1016/S1047-9651(02)00007-4 12380546

[B41] Gilbert JW Martin JC Wheeler GR Storey BB Mick GE Richardson GB Lumbar disk protrusion rates of symptomatic patients using magnetic resonance imaging J Manipulative Physiol Ther 2010 33 626 629 10.1016/j.jmpt.2010.08.010 21036285

[B42] Slavin KV Raja A Thornton J Wagner FC Jr Spontaneous regression of a large lumbar disc herniation: report of an illustrative case Surg Neurol 2001 56 333 336 10.1016/S0090-3019(01)00607-3 11750011

[B43] el Barzouhi A Vleggeert-Lankamp CLAM à Nijeholt GJL Van der Kallen BF van den Hout WB Jacobs WCH Magnetic resonance imaging in follow-up assessment of sciatica N Engl J Med 2013 368 999 1007 10.1056/NEJMoa1209250 23484826

[B44] Jarvik JG Hollingworth W Heagerty PJ Haynor DR Boyko EJ Deyo RA Three-year incidence of low back pain in an initially asymptomatic cohort: clinical and imaging risk factors Spine (Phila Pa 1976) 2005 30 1541 1548 10.1097/01.brs.0000167536.60002.87 15990670

[B45] Stankovic R Johnell O Maly P Willner S Use of lumbar extension, slump test, physical and neurological examination in the evaluation of patients with suspected herniated nucleus pulposus. A prospective clinical study Man Ther 1999 4 25 32 10.1016/S1356-689X(99)80006-X 10463018

[B46] Reihani-Kermani H Correlation of clinical presentation with intraoperative level diagnosis in lower lumbar disc herniation Ann Saudi Med 2004 24 273 275 15387493 10.5144/0256-4947.2004.273 PMC6148114

[B47] Vucetic N Svensson O Physical signs in lumbar disc hernia Clin Orthop Relat Res 1996 333 192 201 8981896

[B48] Kent P Keating JL Classification in nonspecific low back pain: what methods do primary care clinicians currently use? Spine (Phila Pa 1976) 2005 30 1433 1440 10.1097/01.brs.0000166523.84016.4b 15959374

[B49] Cohen SP Gupta A Strassels SA Christo PJ Erdek MA Griffith SR Effect of MRI on treatment results or decision making in patients with lumbosacral radiculopathy referred for epidural steroid injections: a multicenter, randomized controlled trial Arch Intern Med 2012 172 134 142 10.1001/archinternmed.2011.593 22157067

